# Rationale, Design and Methods of the Ecological Study of Sexual Behaviors and HIV/STI among African American Men Who Have Sex with Men in the Southeastern United States (The MARI Study)

**DOI:** 10.1371/journal.pone.0143823

**Published:** 2015-12-23

**Authors:** DeMarc A. Hickson, Nhan L. Truong, Neena Smith-Bankhead, Nikendrick Sturdevant, Dustin T. Duncan, Jordan Schnorr, June A. Gipson, Leandro A. Mena

**Affiliations:** 1 Center for Research, Evaluation and Environmental & Policy Change, My Brother's Keeper, Inc, 510 George Street, Unit 100, Jackson, MS, United States of America; 2 Department of Medicine, University of Mississippi Medical Center, 2500 North State Street, Jackson, MS 39213, United States of America; 3 R.E.D. Institute, AID Atlanta, Inc., 1605 Peachtree Street NE, Atlanta, GA 30309, United States of America; 4 Department of Population Health, New York University School of Medicine, 227 E 30th Street Room 621, New York, NY, 10016, United States of America; 5 Center for Community-Based Programs, My Brother's Keeper, Inc, 710 Avignon Drive, Ridgeland, MS, United States of America; National Center for AIDS/STD Control and Prevention, China CDC, CHINA

## Abstract

**Background:**

This paper describes the rationale, design, and methodology of the Ecological Study of Sexual Behaviors and HIV/STI among African American Men Who Have Sex with Men (MSM) in the Southeastern United States (U.S.; known locally simply as the MARI Study).

**Methods:**

Participants are African American MSM aged 18 years and older residing in the deep South.

**Results:**

Between 2013 and 2015, 800 African American MSM recruited from two study sites (Jackson, MS and Atlanta, GA) will undergo a 1.5-hour examination to obtain anthropometric and blood pressure measures as well as to undergo testing for sexually transmitted infections (STI), including HIV. Intrapersonal, interpersonal, and environmental factors are assessed by audio computer-assisted self-interview survey. Primary outcomes include sexual risk behaviors (e.g., condomless anal sex) and prevalent STIs (HIV, syphilis, gonorrhea, and Chlamydia).

**Conclusion:**

The MARI Study will typify the HIV environmental 'riskscape' and provide empirical evidence into novel ecological correlates of HIV risk among African American MSM in the deep South, a population most heavily impacted by HIV. The study's anticipated findings will be of interest to a broad audience and lead to more informed prevention efforts, including effective policies and interventions, that achieve the goals of the updated 2020 U.S. National HIV/AIDS Strategy.

## Introduction

In the United States (U.S.), disparities in the prevalence and incidence of human immunodeficiency virus (HIV) are broad, substantial, and based on race/ethnicity, gender, sexual orientation/identity and geography [1,2]. African Americans account for more new diagnosed HIV cases than any other racial/ethnic group [3–5]. African Americans comprise approximately one-half (47%) of the new HIV infections that occur in the U.S. each year compared to nearly one-third (28%) for Whites and one-fifth (20%) for Hispanics [6]. African American men, in particular, account for 34% of all incident HIV infections. These stark racial differences are most evident among sexual minority populations, especially gay, bisexual and other men who have sex with men (collectively referred to as MSM herein). African American MSM comprise 39% of new HIV infections among MSM, and the rate of new HIV infections among young African American MSM aged 13–29 years continues to increase [7–9]. Lastly, in terms of geography, the rate of new HIV infections in the U.S. is approximately 18.0/100,000 persons compared to 24.5/100,000 in the deep South [10,11], a region of the U.S. that generally includes six states (Louisiana, Mississippi, Alabama, Georgia, South Carolina and North Carolina) and contains a large portion of African Americans [12] and MSM [13].

There is growing attention, domestically, on HIV among African American MSM in the deep South [14,15] not only because HIV is increasing in prevalence relative to other at-risk groups where the rate either remains static or continues to decline [5–9] but because 1 in 5 African American MSM are living with HIV compared to 1 in 16 Hispanic MSM and 1 in 22 White MSM [7,16]. Surveillance data from the Mississippi State Department of Health reveal a 38% increase in newly diagnosed HIV infections from 2004–2005 to 2006–2007 among young African American MSM in the Jackson, MS metropolitan statistical area (MSA) [17]. In addition, African American MSM account for approximately 25% of the persons living with HIV in the Jackson, MS MSA [18] and data from ongoing epidemiologic and intervention studies estimate the HIV seroprevalence to be 25–35% [19,20]. In Georgia, surveillance data from the Georgia Department of Public Health indicate that men account for three quarters (74%) of all new diagnosed HIV cases and approximately 40% of these cases are due to male-to-male sexual contact [21]. Cohort studies among African American MSM in the Atlanta, GA MSA, suggest that HIV seroprevalence to be 20–45% [22–24]. Taken together, these data highlight that the rates of HIV among African American MSM in Mississippi and Georgia are amongst the highest in the nation [7,8] and that public health researchers must intensify their efforts to identify relevant determinants that may mitigate HIV acquisition and transmission in this high-risk population in order to achieve the goals of the updated 2020 U.S. National HIV/AIDS Strategy [25].

Although there has been a wealth of research into HIV among MSM populations overall, relatively few epidemiologic cohort studies include large samples of African American MSM [22–24], and even fewer specifically focus on African American MSM in the deep South [22]—the epicenter of the U.S. HIV epidemic [10,11]. Existing behavioral research shows that African American MSM are just as likely as White MSM to engage in condomless anal sex, sex with a known HIV-positive partner and sex for goods and services, while on the other hand, African American MSM report fewer sex partners than White MSM [26,27]. However, these sexual risk behaviors do not explain the disproportionate rates of HIV in African American MSM. Studies of individual-level, or proximal, factors report that depressive symptoms [28], internalized homophobia [29,30], low optimism [31], and role flexing/masculinity [29,32] are directly or interactively associated with HIV risk among African American MSM. Other studies that consider macro-level, or distal, factors (e.g., interpersonal, neighborhood or political) demonstrate that early life sexual traumas [33], forced sexual experiences in adulthood [34], experiences of homophobia and discrimination [29,35], and social norms that support high-risk sexual behaviors [36,37] are associated with HIV sexual risk and testing behaviors, as well as beliefs of HIV vulnerability among African American MSM. In addition, evidence from sexual network studies reveal that multiple contexts within the sexual dyad, including having commercial (e.g., exchange or anonymous) sex partners and having sex early upon meeting sexual partners, are associated with increased levels of HIV acquisition and transmission behaviors among African American MSM [38–40]. Moreover, researchers report that having older and African American male sexual partners account for 20% of the African American-White disparity in HIV prevalence [41] whereas experiences of discrimination (sexual orientation and race-based) and resiliency do not explain any of the disparity between African American and White MSM in Atlanta, GA [42]. Finally, an emerging literature suggests that structural stigma, neighborhood residential segregation, and other neighborhood-level characteristics are associated with HIV risk among African American MSM [43–45].

The Institute of Medicine recommends the prioritization of HIV prevention research among MSM to utilize theoretical frameworks such as the socio-ecological model to fully characterize the environmental 'riskscape' (i.e., the set of individual, interpersonal, environmental and political factors that increase the susceptibility of disease [46]) of MSM [47]. The socio-ecological model is a promising multi-level approach that considers the complex interplay between individual (or intrapersonal), interpersonal, environmental (e.g., neighborhood), and political factors and their collective contribution to the etiology of HIV among MSM [48]. As such, the Ecological Study of Sexual Behavior and HIV/STI (sexually transmitted infections) among African American MSM in the Southeastern U.S. [known locally simply as the MARI Study (because of the long study title and has no acronymic definition), but should not be confused with the Minority HIV/AIDS Research Initiative (MARI) from the Division of HIV/AIDS Prevention at the Centers for Disease Control and Prevention (CDC)] will typify the HIV environmental 'riskscape' of African American MSM in the deep South and examine the direct and indirect associations of these multi-level contexts in relation to HIV risk. Specifically, using a socio-ecological perspective as recommended by the IOM, the specific aims of the MARI Study are to:

1. explore the interrelationships between socio-demographics, condom self-efficacy, psychosocial factors and personality traits, and body image/anthropometrics (intrapersonal); early life circumstances and family dynamics, perceived experiences of discrimination, stress from major life events, social support, and peer norms about condoms (interpersonal); and, gay-friendly neighborhoods and other neighborhood-level characteristics (environmental);
2. examine the cross-sectional associations of individual, interpersonal, and environmental factors, and their interactive effects, with sexual risk behaviors and HIV/STI infection; and
3. investigate geographic (e.g., urban versus rural) differences in the associations in Aim 1 and Aim 2.

This paper describes the design, sampling methods, and data collection and management methods of the ongoing MARI Study.

## Materials and Methods

### Study Participants and Sites

The MARI Study is a collaboration between My Brother's Keeper, Inc and AID Atlanta, two community-based organizations (CBOs) with nearly two decades of experience addressing HIV in African American communities in Jackson, MS and Atlanta, GA, respectively. The research visits occur at a single study site (Open Arms Healthcare Center in Jackson, MS and AID Atlanta Health Services Clinic in Atlanta, GA) located near the geographic center of each MSA. The Open Arms Healthcare Center (www.oahcc.org) is a community-based, primary care clinic focused on the health needs of the lesbian, gay, bisexual and transgender (LGBT) community and other underserved populations [49]. The AID Atlanta Health Services Clinic (www.aidatlanta.org) is a primary care clinic that provides comprehensive medical services to persons living with HIV. Thus, these clinics serve as primary portals for the medical care of underserved and highly marginalized populations, including African American MSM.

The population source for the MARI Study is population-based African American MSM in Jackson, MS and Atlanta, GA. To adequately typify the HIV environmental 'riskscape' and to evaluate the potentially salient multi-level HIV risk and protective factors among African American MSM, the study will enroll 800 African American MSM to adequately power the aforementioned specific aims. In particular, the study is designed, *a priori*, to enroll approximately equal numbers of African American MSM from the two study sites. Therefore, each study site will enroll 400 African American MSM.

Participants at the Jackson, MS site are recruited from urban and rural areas in the five-county MSA of Copiah, Hinds (which includes the city of Jackson, the capital and largest city in the state of Mississippi), Madison, Rankin, and Simpson counties. The Jackson, MS MSA, geographically, is made up of 2,425.40 square-miles in land area and contains approximately 500,000 people, over half (260,000) of whom are African American [50]. Nearly 20% (19.7%) of the population in the Jackson, MS MSA lives below the poverty level and has less than a high school education; the per capita income in the Jackson, MS MSA is $23,429 [50].

Similarly, participants at the Atlanta, GA site are recruited from the five-county MSA of Clayton, Cobb, Dekalb, Fulton (which includes the city of Atlanta, the capital and largest city in the state of Georgia), and Gwinnett counties. The Atlanta, GA MSA, is comprised of a land area of 6,207.94 square-miles and contains an estimated 5,270,000 people, one-third (1,708,000) of whom are African American [50]. The per capita income among adults in the Atlanta, GA MSA is $28,504 and approximately 18% of the population lives below the poverty level and roughly one in ten has less than a high school education [50].

According to Lieb et al (2009), approximately 10,200 and 50,000 African American MSM reside in the states of Mississippi and Georgia, respectively. Given the demographic composition of the Jackson (18.3% of the state's population) and Atlanta (35.4% of the state's population) MSA as the largest geographic areas in each respective state, these catchment areas provide adequate sources of African American MSM (Jackson: 1,900 and Atlanta: 17,700) to recruit. In addition, the 2012 CDC HIV Surveillance Report indicates that Jackson, MS and Atlanta, GA account for roughly six percent of all new HIV infections in the U.S. [5], and thus, are geographic areas with adequate sources of African American MSM at increased risk for HIV infection.

### Eligibility

Individuals must self-identify as African American or Black and report residence in the Jackson, MS or Atlanta, GA MSA to be eligible for the study. Inclusion criteria further require a self-report of (1) assigned biological male sex at birth; (2) being 18 years or older; and, (3) engaging in oral or anal sex with another man in the six months prior to study enrollment (Table 1). This latter eligibility criteria includes MSM who have sex with women as well as MSM who have sex with men only.

**Table 1:** The MARI Study Inclusion and Exclusion Criteria.

**Inclusion Criteria**
Age of 18 years or older
Self-identification as African American or Black
Male sex at birth
Residence in either the Jackson, MS or Atlanta, GA metropolitan statistical area (MSA)*
Able to speak and read English
Willingness to provide written informed consent
**Exclusion Criteria**
Age under 18 years of age
Women
Men who report have sex with women exclusively
Individuals unable to speak or read English
Individuals unable or not willing to provide written informed consent
Residence outside of either the Jackson, MS or Atlanta, GA MSA
Medical, psychiatric or behavioral factors that in the judgment of the principal investigator or trained study personnel may interfere with study participation
Inability to follow the requirements of the study protocol

*Jackson, MS MSA = Copiah, Hinds, Rankin, Madison and Simpson counties; Atlanta, GA MSA = Clayton, Cobb, Dekalb, Fulton, and Gwinnett counties.

### Recruitment

Recruitment will occur between 2013 (July 2013 at the Jackson site and January 2014 at the Atlanta site) and 2015 (until the aforementioned recruitment targets are reached or December 31, 2015). Building on our experience addressing HIV among African American MSM in our current and past portfolio, we utilize a variety of population-based sampling methods in the current study. Similar to prior work [51–54], participants are recruited from the community through (1) the distribution of printed advertisements at local colleges and universities, adult bookstores, bars and clubs, as well as CBOs servicing African American MSM; (2) face-to-face recruitment from local bars and clubs frequented by African American MSM, and HIV prevention interventions, community events, and other activities conducted by the partnering CBOs; (3) social networking websites/applications ('apps'), including Facebook and Twitter; (4) geospatial sexual networking 'apps', including Jack'd and Grindr; and (5) word-of-mouth referrals.

First, research staff obtain permission from the appropriate college/university administrator (e.g., Dean of Student Affairs) and local adult bookstore, bar, and/or club owner to distribute pushcards to students, faculty and other staff (on the campuses of the local colleges and universities) or bookstore, bar and/or club patrons. Research staff also obtain permission to post recruitment posters with perforated strips containing study contact information, which can be torn off, in specified areas at the venue/institution. Second, face-to-face recruitment is conducted at local venues (bars and clubs) frequented by African American MSM and during HIV prevention interventions and testing events, and other various community events hosted by the local CBOs. Importantly, research staff wear promotional t-shirts that contain details about the study to increase visibility and garner interest in the study. Third, social and geospatial networking 'apps' are utilized to recruit study participants. An account/profile is created on each site/'app' and serve as a portal to communicate with and screen potential participants. A recruitment script, with probes, gives research staff flexibility to communicate with potential participants, and thereby develop a rapport and sense of trust with these individuals. The main theme of the script focuses on improving the overall health of African American men. Finally, word of mouth referrals such as from participants who have already completed the study, local agencies and organizations that serve African American MSM, and clinical staff at Open Arms Healthcare Center and AID Atlanta Health Services Clinic, are utilized to recruit study participants.

### Screening Procedures

Research staff complete an eligibility screening questionnaire validated using two focus groups containing nine African American MSM prior to study initiation (February 2013) to screen interested individuals for study eligibility (See Table 1). Individuals who screen eligible are scheduled an appointment [at Open Arms Healthcare Center (Jackson, MS) or AID Atlanta Health Services Clinic (Atlanta, GA)] in two-hour appointment blocks to allow time for completion of all study procedures and administrative tasks (e.g., informed consent process). Reminder calls or text messages are made the day before the scheduled appointment as well as at least one hour before the scheduled appointment to confirm or reschedule the appointment. Additionally, since transportation is often a barrier to participation in HIV prevention services among African American MSM [55], participants are offered transportation services to the study site at no cost. In Jackson, MS, Open Arms Healthcare Center or the research staff provide transportation services for potential participants to and from the study site. Upon pickup, the potential participants sign a waiver that releases My Brother's Keeper, Inc from any liability for injury and/or death that may occur as a result of the transportation. In Atlanta, GA, MARTA cards (public transportation system that covers the Atlanta, GA MSA) are given to eligible individuals who express transportation needs.

### Ethics Approval

The Sterling Institutional Review Board reviews and approves the MARI Study, according to federal regulations for research with human subjects. A Certificate of Confidentiality from the CDC protects the privacy of research study participants against compulsory legal demands, such as court orders and subpoenas, as participants report on potentially illegal behaviors. At the time of enrollment into the study, participants are informed of the Certificate of Confidentiality.

### Overview of the Study Visit

The study visit includes several health screenings and a study questionnaire, which lasts approximately 1.5 hours and begins with the informed consent process. The informed consent process takes place at each study site in a private interview room by research staff specifically trained in this process. Participants provide written informed consent and are given a copy of the consent document for their record. After providing informed consent, participants undergo various health screenings that include anthropometry (Tanita Body Composition Analyser TBF-300A; Tanita Corporation, Japan, wall-mounted stadiometer; Seca 222, Seca GmBH & Co., Germany, and Gulick anthropometric tape; Creative Engineering, Plymouth, Michigan), seated blood pressure (IntelliSense Professional Digital Blood Pressure Monitor HEM-907XL; OMRON Corporation, Japan); phlebotomy for syphilis (BD Macro-Vue™ RPR Kits, Maryland, USA); and HIV (Clearview Complete HIV ½ Rapid Test; Alere, U.S.A), Chlamydia and gonorrhea testing (Aptima CA2, Hologic, San Diego, California). After the health screenings, participants complete a study questionnaire. The procedures for these study components are described in greater detail below.

#### Anthropometry and Blood Pressure

After informed consent, participants are escorted to a private restroom and instructed to change into a light-weight surgical scrub suit provided by each study site. Each study site provides participants with a safe place to store clothing and valuables. Anthropometry is performed by research staff and includes standing height, standing weight, body composition measurements (including body mass index, % body fat, and muscle mass), and circumferences of the neck, chest, biceps (left and right), waist and hip. After a five minute wait in a quiet room, research staff ascertain two blood pressure (systolic and diastolic) and pulse rate measurements, taken one minute apart, from the right arm of seated participants whose back and arm were supported using an appropriately sized cuff. Research staff collecting anthropometric and blood pressure measures are non-clinical professionals who have been trained by a registered nurse with more than 20 years of nursing experience and experience working with cohort studies such as the Jackson Heart Study.

#### HIV/STI Testing

**Fig 1:**
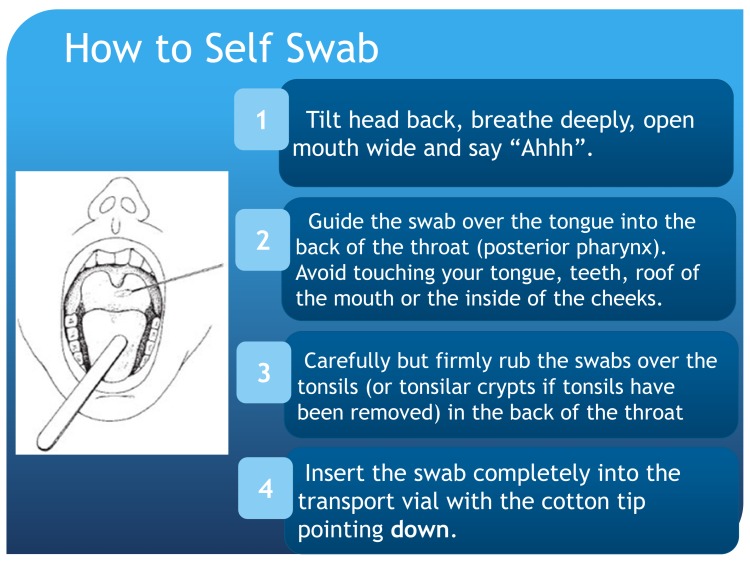
Instructions, with Illustrations, for the Self-Collected Pharyngeal Swab for Gonorrhea and Chlamydia Testing (Aptima CA2, Hologic, San Diego, California). The MARI Study.

**Fig 2:**
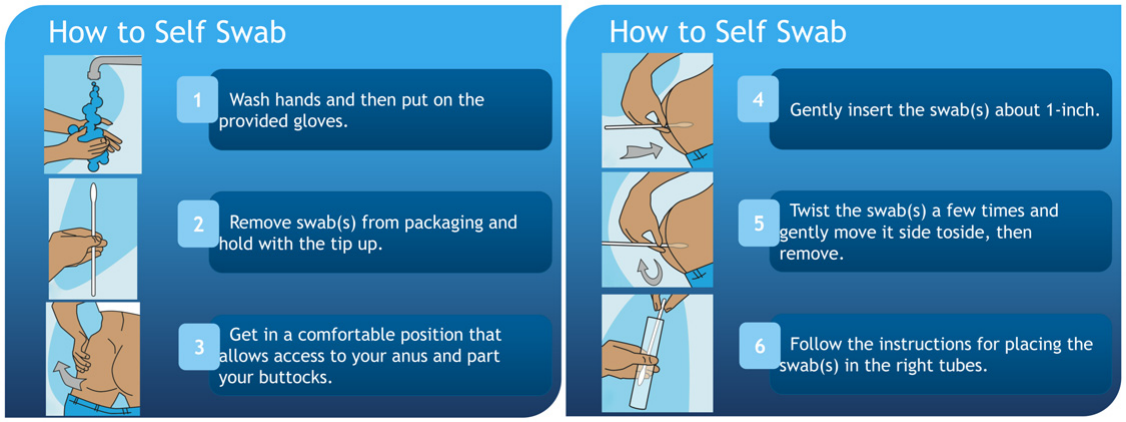
Instructions, with Illustrations, for the Self-Collected Rectal Swab for Gonorrhea and Chlamydia Testing (Aptima CA2, Hologic, San Diego, California). The MARI Study.

Research staff certified in HIV counseling, testing and referral first administer a paper-based questionnaire to ascertain a history of HIV and syphilis infection. Accordingly, research staff perform rapid HIV testing after risk reduction counseling for HIV-uninfected participants or participants unaware of their status. Participants aware of their HIV infection sign an Authorization for the Release of Health Information form, which allows the PI to obtain HIV infection information such as date of infection, engagement in care, CD4 count and viral load for the past 12 months. Next, whole blood is collected from each participant using a Vacutainer blood collection tube (venipuncture) for syphilis testing. After venipuncture, participants are escorted to a private restroom and instructed on the self-collection of pharyngeal and rectal swabs as well as a urine specimen for gonorrhea and Chlamydia testing. Instructions, with illustrations, are posted in visible areas in the restroom at each study site (Figs 1 and 2). Prior work among MSM shows that self-collected pharyngeal and rectal swabs are equally reliable in detecting gonorrhea and Chlamydia infections as compared to swabs collected by trained health professionals [56].

#### Study Questionnaire

After participants remove the scrub suit and redress in their own attire, participants are accompanied to a private interview room to complete a study questionnaire. The list of study measures contained in the study questionnaire is provided in Table 2. We selected measures that capture the multi-level constructs necessary to typify the HIV environmental 'riskscape' of African American MSM and to address our primary study aims, including the potential confounders or effect modifiers of our hypothesized associations. All questions have been pilot tested using two focus groups with 9 African American MSM in each group prior to study initiation (February 2013) and revised accordingly prior to administration in the study. With the exception of the HIV and syphilis history survey (as this survey is administered in the laboratory prior to HIV/STI testing), all questions are administered via an audio computer-assisted self-interview (ACASI) survey. An ACASI was chosen as the method of administration (versus interviewer-administration) in order to decrease the misclassification of sensitive questions such as experiences of sexual abuse in childhood and under-reporting of socially undesirable behaviors such as condomless anal sex [83].

**Table 2:** A socio-ecological model perspective. Multi-level influences of HIV risk among African American MSM in The MARI Study.

Levels	Measures
**Intrapersonal: Individual characteristics such as knowledge, attitudes, and beliefs, psychosocial factors, and personality traits that influence behavior**	
Primary Outcomes	Prevalent HIV and STI*,**; Sexual risk behaviors**
Socio-demographics, health insurance status, substance abuse, and history of incarceration and homelessness	Date of birth, sexual orientation, gender non-conformity [57], educational attainment, annual household income, subjective social status [58], healthcare access and utilization, circumcision status, alcohol and drug use, history of incarceration and homelessness.
Anthropometry*	Height and weight; Bicep, chest, waist and hip circumferences; and Percent body fat and percent muscle mass
Blood pressure*	Systolic and diastolic and pulse (beats per minute)
Mental health	Depressive symptomatology [59]
Body image	Body image, including appearance evaluation and orientation and overweight preoccupation [60], social physical anxiety [61], and figure (body shape) rating [62].
Personality traits	Anger [63], hostility [64], optimism [65], and resilience [66]
Religiosity and Spirituality	Organized and non-organized religion, religious coping, and daily spiritual experiences [67]
**Interpersonal: Social interaction processes and primary groups, including family dynamics, social networks and situational contexts.**	
Early life environment	Early life sexual experiences [68] and adverse circumstances in childhood [69]
Social and minority stressors	Experiences of discrimination [70], discrimination due to sexual orientation and HIV-status [71], major life events [72]
Social support	Multi-dimensional social support and social network [73,74]
**Community: Neighborhood characteristics that influence one's exposure to health-promoting or health-damaging resources.**	
Residential history	Geocoding/GIS, Residential mobility
Neighborhood Environment	Social cohesion [75], disorder [76,77], safety and violence [78,79], neighborhood disadvantage [80–82], LGBT-friendliness

Abbreviations. MSM = Men who have sex with men; HIV = Human immunodeficiency virus; STI = Sexually transmitted infections. LGBT = Lesbian, gay, bisexual, and transgender; GIS = Geographic Information System

*Measures were not part of the audio computer-assisted self-interview survey.

**STIs include gonorrhea and Chlamydia (rectal, urethral, and pharyngeal) and Syphilis

**Sexual risk behaviors include condomless anal, vaginal and oral sex, condom use, and multiple sexual partners.

At the end of the study visit, research staff perform an exit interview to provide participants with a brief interpretation of their anthropometric and blood pressure measurements and (preliminary) HIV test result as well as, if applicable, to ensure completion of the study questionnaire and confirm the participant's contact information for delivery of their STI test results. Participants are compensated $35 (which later increased to $50, as approved by the Sterling IRB, in order to improve study participation) for their time and given a bag of complimentary study promotional items, including a study-related t-shirt, lanyard, bracelet and pen, and 30-day supply of quality latex condoms and water-based lubricants.

As appropriate, participants with a reactive HIV test are accompanied to the respective clinic (Jackson, MS: Open Arms Healthcare Center; Atlanta, GA: AID Atlanta Health Services Clinic) at the conclusion of the study visit for confirmatory testing. This ensures that all participants with a reactive HIV test are linked to the appropriate care services as each study site is a primary healthcare facility that provides a range of HIV care services and maintains a referral network of other community social and clinical resources.

### Selection of Measures

The selection of study measures is guided by the socio-ecological model for MSM [48], which provides a multi-level framework of proximal and distal determinants of HIV risk. Importantly, the socio-ecological model framework allows for the inclusion of micro (e.g., depressive symptoms, resilience) and macro-social (childhood sexual abuse, neighborhood gay friendliness) factors that serve as regulators, either directly or interactively, of HIV risk. Sexual risk behaviors and prevalent HIV and STIs (Chlamydia, gonorrhea and syphilis) have been selected as the primary outcomes because they facilitate the acquisition and transmission of HIV. Sexual risk behaviors include alcohol and/or drug use before or during sex, condom use, condomless anal, vaginal, and oral sex with casual and main partners, and multiple sexual partners.

Many of the study measures have been validated for use among African American MSM in previous studies. However, when validated measures are not available, we revised existing measures for use in the current study. We plan to develop separate manuscripts that will describe and document the psychometrics of several socio-cultural measures (e.g., Daily Spirituality Experiences Scale and Everyday Discrimination Scale) that have not been previously measured in African American MSM. Residential mailing addresses (for the past five years) are ascertained from study participants and will be retrospectively geocoded [84,85]. This will allow us to develop an innovative measure of mobility as well as link to publically and commercially-available neighborhood-level measures such as neighborhood disadvantage [81,82] and LGBT hate crimes [86] to study data, which is a major strength of the study.

### Central Laboratory

The MSDH Public Health Laboratory serves as the central laboratory and maintains a protocol for specimen collection, storage and transport. Urine and blood specimens and pharyngeal and rectal swabs (including those shipped to My Brother's Keeper from the Atlanta site via FedEx using the appropriate biohazard packaging) are hand-delivered weekly to the MSDH Public Health Laboratory. The Georgia Public Health Laboratory accepts and processes blood specimens (for syphilis testing) from the Atlanta study site. All results, irrespective of study site, are posted on a secure encrypted online system within seven days of delivery to the respective laboratory.

### Data Management, Quality Assurance and Control, and Statistical Analysis

My Brother's Keeper, Inc's Center for Research, Evaluation and Environmental & Policy Change is the study's coordinating center and is responsible for the data management, quality assurance and control procedures, and data analyses for the study. In collaboration with the Atlanta site research team, The MARI Study Coordinating Center maintains a comprehensive data capture and management system along with quality assurance and control procedures to ensure rigorous and high-quality data collection and adherence to study protocols. With the exception of the ACASI, data collected via paper-based forms are manually entered into standardized databases [Microsoft Office Excel 2007, Redmond, WA] and are transmitted to The MARI Study Coordinating Center via a secure and encrypted email system. In addition, all forms, including original copies of the Informed Consent Form, from the Atlanta study site are shipped via FedEx to The MARI Study Coordinating Center for the proper data storage and recordkeeping.

Data collection and entry are important processes to ensure good quality data, which lead to sound and reliable conclusions. After the completion of each participant study visit, the Quality Assurance Manager at each study site reviews each participant's study folder for completion and notes any missing data components or changes in the order of the study procedures (e.g., perform urine collection immediately after the informed consent process due to a participant's need to urinate). The participant study folder is then given to the appropriate research staff to address/update the missing data components or for data entry. Double data entry [87] is utilized to identify systematic errors in manually entered data. Therefore, a second research staff member periodically enters data collected on paper-based forms (e.g., anthropometry and HIV/Syphilis History Form) from 10% of the sample for quality control purposes. Additionally, the data management team performs several quality control checks (e.g., digit preference for anthropometric circumference measures) and communicates important discrepancies to the PI. Finally, The MARI Study Coordinating Center guides the analytics of the study, which will include calculating prevalence estimates of primary outcomes, typifying the HIV environmental 'riskscape', and addressing the aforementioned Specific Aims.

### Dissemination Plan & Data Sharing

It is anticipated that novel and unparalleled findings will result from The MARI Study and will have broad scientific interest and immediate public health applicability. The research team maintains an intricate plan to disseminate study findings to both professional and lay communities as well as circulate study data to study collaborators. The dissemination plan includes presentations in professional and community meetings (e.g., meetings of the American Public Health Association), publication of scientific manuscripts in peer-reviewed journals, and lay summaries/fact sheets of published work circulated to study participants and local agencies that serve African American MSM. Finally, every effort will be made to make these data accessible for research purposes, which includes the development of a Research Writing Group that currently meets on a bi-weekly basis to discuss preliminary study results, develop concept sheets for additional scientific manuscripts, and future research ideas. Study investigators also maintain a Data Distribution Agreement in order to protect the privacy and confidentiality of participants and their families. Both Internal and External researchers and collaborators interested in accessing study data may contact the PI (Hickson) and must agree to adhere to the requirements of the Data Distribution Agreement.

## Discussion

The MARI Study will be one of the largest and most robust cohort studies into the etiology of HIV among African American MSM in the deep South. The MARI Study will recruit 800 African American MSM in the deep South to explore the interrelations between early life circumstances, familial dynamics, and socio-cultural (including neighborhood) contexts and their direct and interactive associations with sexual risk behaviors and HIV/STI seroprevalence. Researchers purport that the African American-White HIV disparity will continue because of disparities across the HIV care continuum [88]. However, evidence from large-scale epidemiologic studies that consider novel theoretical frameworks to understand the multi-faceted and multi-level exposures that contribute to HIV outcomes among African American MSM is lacking [22–24]. Equally, the ever-expanding HIV epidemic experienced by African American MSM, especially African American MSM in the deep South, has not been matched by equal attention in epidemiologic research that explores the multiple pathways beyond individual-level risk behaviors that may explain variations in HIV among MSM populations [89].

To address this knowledge gap, The MARI Study will typify the HIV environmental 'riskscape' and provide evidence into the ecological correlates of HIV for African American MSM in the deep South, the population most heavily impacted by HIV in the U.S. [7–9]. These new and unprecedented findings will largely inform prevention efforts and opportunities to intervene to reduce the African American-White HIV disparity. For example, if African American MSM in The MARI Study report high levels of resilience, as measured by the Connor-Davidson Resilience Scale, and if those with the highest scores engage in less HIV transmission behaviors (i.e., condomless anal sex) then public health practitioners can develop high-impact prevention strategies that promote individual, social and structural resilience such as affirming messaging that eliminates discrimination (race and sexual orientation) and stigma (HIV-serostatus). Second, our work will have policy relevance: increasing efforts to combat incarceration rates among young African American MSM through the creation of educational training programs that reduce high school dropout rates and enforcement of laws that limit policing of LGBT or youth of color. Finally, we will be able to identify geographic locations for HIV prevention interventions, including geographically targeted HIV testing, as we begin to document relationships between environmental characteristics and HIV risk. Therefore, evidence from this study will provide unequivocal evidence into the underlying mechanisms of HIV in African American MSM and inform a variety of prevention efforts and effective federal, state, and local policies for eliminating disparities in this high-risk population. Notable strengths of The MARI Study include population-based sampling to recruit participants, a multi-site design, and a multi-level characterization of the HIV environmental 'riskscape' for African American MSM in the deep South. This approach will allow us to tease apart the complex interplay between individual, interpersonal and neighborhood-level factors and their concomitant influence on HIV risk in this highly marginalized population. Also, our study is highly efficient from the perspective of recruiting a hard-to-reach population [90,91] due to the inclusion of two CBOs (My Brother's Keeper and AID Atlanta) each with over 15 years of experience addressing HIV among African Americans MSM. Finally, the inclusion of novel individual (e.g., resilience, optimism), interpersonal/social (e.g., risky early life circumstances) and neighborhood (e.g., gay friendliness of neighborhood) risk and protective factors will help to identify relevant constructs in future papers that may explain the African American-White disparity, especially as national public health agendas attempt to improve access to healthcare for marginalized populations (e.g., through the Affordable Care Act) [92].

The MARI Study has some limitations. First, the current study is specific to African American MSM in two MSAs in the deep South; therefore, findings may not be generalizable to other geographically placed African American MSM as the current study was not designed to be representative of African American MSM in this region of the U.S. However, the extraordinarily high prevalence of HIV among African American MSM in the deep South [7–9] warrants the initiation of large-scale epidemiologic studies such as The MARI Study to investigate the underlying causes of disease etiology in African American MSM and to learn how best to prevent HIV in this population in the future. The design of The MARI Study is cross-sectional, so this limits our ability to infer causal inferences and how risk and protective factors are associated with HIV infection over time. In anticipation of funding for follow-up studies including repeated examinations, we developed several retention procedures, including the collection of detailed contact information, to follow participants more effectively as well as the study design and operations will allow for prospective investigations into incident HIV and other STIs. To date, over 97% of study participants have indicated interest in participating in future research studies such as the MSM Identities Study [93], an ancillary study designed to understand the diversity of identities, practices, and sexualities among African American MSM in the Jackson, MS area.

The data and materials produced by The MARI Study are intended as a resource to the scientific community and will be accessed by many researchers through mechanisms that protect and serve the interests of the African American MSM communities in the Jackson and Atlanta MSAs. The careful and robust use of this resource will provide substantial benefits to these communities and will ultimately inform bio-behavioral prevention efforts that eliminate longstanding and pervasive HIV health disparities among African American MSM in the deep South.
